# Current Trends in Antifungal Prophylaxis for High-Risk Neonates in Neonatal Intensive Care Units in India: A Nationwide Survey

**DOI:** 10.7759/cureus.36136

**Published:** 2023-03-14

**Authors:** Sumitha Arun, Mereta Varghese, Taliya Cherian, Prakash Ramaswami

**Affiliations:** 1 Neonatology, Believers Church Medical College Hospital, Thiruvalla, IND; 2 Biostatistics, Christian Medical College Vellore, Vellore, IND

**Keywords:** nicu, fluconazole, preterms, neonates, antifungals

## Abstract

Background

Prophylactic antifungals are often used in high-risk babies in neonatal intensive care units (NICUs) to reduce invasive fungal infections (IFIs). However, existing guidelines regarding prophylactic antifungal usage do not clearly define the high-risk population. This study aimed to assess the practices related to prophylactic antifungal use in NICUs in India.

Methods

For this cross-sectional study, an online structured questionnaire was completed by neonatologists who worked in level 3 NICUs in 12 states in India during the period June 2022 to August 2022.

Results

Data from 151 NICUs were analyzed. 71.7% of respondents were from private hospitals, and 28.3% were from government hospitals. Nearly one-fourth of the units (28.5%) used antifungal prophylaxis in all extremely low birth weight (ELBW) babies, while another one-fourth (25.8%) used a case-based approach. The remaining NICUs (45.7%) did not use prophylactic antifungals. Among the users, the preferred antifungal was fluconazole; 3 mg/kg and 6 mg/kg were the dosage regimens used. The commonly used interval for administering fluconazole was 72 hours (69.2% of units). The intravenous route was the preferred route of administration (84.1%). Factors that influenced the non-users were the low incidence of fungal infections in their NICUs and concern about the development of resistance. It was noted that the users felt strongly about the need for further recommendations from pediatric societies and more studies on the efficacy of antifungals.

Conclusion

There is considerable variation in the use of prophylactic antifungals across NICUs in India. Among the users, uniformity in the choice of antifungal and interval of administration was observed. Further recommendations from pediatric societies, including the definitions of neonates at-risk of fungal infections, are required to ensure consistency in practice and help clinicians decide whether or not to use prophylactic antifungals.

## Introduction

Invasive fungal infections (IFI) are serious illnesses that affect preterms and high-risk neonates. The majority of these infections are caused by Candida albicans and Candida non-albicans, like Candida parapsilosis. The symptoms are non-specific, and the identification of the organism on a blood culture can be difficult in preterm babies. The diagnosis can therefore be delayed. These infections are known to cause significant mortality and morbidity [1]. The mortality rate before discharge for babies with birth weight less than 1000 g and invasive candidiasis (IC) is 30-40%. Follow-up data for extremely low birth weight (ELBW) babies with IC at 18-22 months showed that 73% of children died or had neurodevelopmental impairment [2,3]. Hence, neonatal intensive care units (NICU) often adopt several preventive strategies. The use of antifungal agents as a prophylactic measure is one such strategy.

The success of using prophylactic antifungals to significantly reduce IFI is unclear. A meta-analysis including 1690 infants from 15 studies showed that only those NICUs with a high incidence of fungal infections showed a reduction in IFI with routine use of antifungal prophylaxis. There was no significant reduction in mortality [4]. This unclear benefit of using prophylactic antifungals in NICUs has led to wide variations in practice.

The variation in practice among neonatologists was observed in previous studies from Japan and Europe. In a nationwide survey from Japan, it was noted that only 43% of all NICUs used antifungal prophylaxis [5]. The results from a similar European study showed that 55% of NICUs used prophylaxis [6]. However, similar data from India is lacking. This survey aimed to study the practice of using antifungal prophylaxis in NICUs in India.

## Materials and methods

This questionnaire-based cross-sectional study was conducted between June and August 2022 among level 3 NICUs in India. Level 3 NICUs are defined as those providing care to babies with birth weights less than 1 kg. The study included all the level 3 NICUs in 12 pre-identified states in India. Two states from each of the six geographical zones of India were selected for the purpose. They were Punjab and Delhi (north zone); Kerala and Tamil Nadu (south zone); West Bengal and Orissa (east zone); Rajasthan and Maharashtra (west zone); Madhya Pradesh and Uttar Pradesh (central zone); and Tripura and Assam (northeast). There is no centralized registry in India, so the NICUs in the 12 states were identified through the National Neonatology Forum office bearers.

Ethical committee approval was obtained from the Institutional Review Board at Believers Church Medical College Hospital, Thiruvalla, India (IEC/2022/09/282). Based on a previous survey from Japan, where 43% of NICUs used routine antifungal prophylaxis with a precision of 20%, the required sample size was 148. A questionnaire was sent by phone or email to one consultant from the NICU. The first and second reminders were sent at seven-day intervals. The first part of the questionnaire dealt with the use of antifungals in the NICU. The second part was about factors that influenced the unit’s practice regarding antifungal use. We have used the same nine factors that were studied in a 2004 US survey and a 2010 European survey on factors that influenced clinicians' decisions regarding prophylactic antifungals [6,7]. The response was measured on a Likert scale and scored from 1 to 5 (≤3 as less important and ≥4 as most important). We performed all analyses using R 4.1.2 (RStudio, Boston, MA). Descriptive data are expressed as frequency and percentage for categorical variables. To determine a significant association between the factors assessed and the clinician’s decisions, a chi-square or Fisher’s exact test was used (significance level of 5%).

## Results

The survey response rate was 63%, and responses from 151 units were collected. Most of the respondents were from private institutions (71.7%), as compared to government hospitals (28.3%). Of the respondents, 28.5% (43/151) of the units use antifungal prophylaxis for all ELBW babies, and 25.8% (39/151) use a case-based approach (Table 1).

**Table 1:** Use of antifungal prophylaxis in neonatal intensive care units in India NICU: neonatal intensive care units

Use of prophylactic antifungals in NICU	Frequency	Percentage
			Yes	43	28.5
Case-based	39	25.8
No	69	45.7
Total	151	

Fluconazole was the preferred agent in 79 of the 82 units using antifungal prophylaxis (96%). The most commonly used dosage regimens of fluconazole were 3 mg/kg and 6 mg/kg (44.3% each), and 11.4% opted for other regimens. Regarding the frequency of drug administration, 69.2% of the users used fluconazole at an interval of 72 hours, 18.7% used it every 24 hours, and 9.3% every 48 hours. The intravenous route (84.1%) was preferred to oral (9.3%), and others (6.6%) based their decision on the availability of intravenous access.

Of the 82 units that used antifungal prophylaxis, the common indications were very low birth weight babies (43 units, 52%), central line in-situ (34 units, 41%), and use of total parenteral nutrition >5 days (27 units, 33%) (Table 2).

**Table 2:** Indications for antifungal prophylaxis in Indian neonatal intensive care units

Indications	Frequency (percentage) n=82
Very low birth weight babies	43(52%)
Central venous line in-situ	34(41%)
Total parenteral nutrition >5 days	27(33%)
Prolonged antibiotics	23(28%)
Abdominal surgery	13(16%)

The criteria for discontinuing antifungals were a predetermined number of days (30.4%), availability of intravenous access (18%), target gestational age (9%), target weight (7.6%), and others (35%). When amphotericin B was used for the treatment of invasive fungal infections, the conventional preparation of amphotericin B was preferred by 45% (68/151) of units and the liposomal preparation by 36% (55/151 units); 19% of the NICUs used both formulations equally.

The key factors that influenced the clinicians' decision on whether to use prophylaxis or not were the incidence of candidiasis, the emergence of resistance, and guidelines from pediatric societies on high-risk neonates requiring prophylaxis. There were significant differences between the users and non-users. The non-users were strongly influenced by the low incidence of candidiasis in the NICUs. As compared to non-users, the users endorsed the need for recommendations from pediatric societies and additional studies on the efficacy of drugs. The cost and safety profile of antifungals were not of significant concern to the respondents (Table 3).

**Table 3:** Factors that influenced the clinicians’ decision regarding the use of prophylactic antifungals in the neonatal intensive care units

Antifungal prophylaxis	Users		Non-users		p-values
Factors that affected clinicians' decision	Likert ≤ 3	Likert ≥ 4	Likert ≤ 3	Likert ≥ 4	
The incidence of candidiasis in your NICU is(or)is not high enough to justify prophylaxis	19 (45)	23 (55)	29 (27)	78 (73)	0.0273
Widespread antifungal use could lead to increased antifungal resistance	12 (29)	30 (71)	16 (15)	91 (85)	0.0867
Statement by paediatric societies in support of routine use in a subset of newborns is needed	8 (19)	34 (81)	57 (53)	50 (47)	0.0003
The criteria of high-risk patients in whom prophylaxis should be attempted need clarification	10 (24)	32 (76)	32 (30)	75 (70)	0.3697
The agent is too costly	34 (81)	8 (19)	91 (85)	16 (15)	0.5654
The role of surveillance culture in identifying high-risk neonates needs clarification	19 (45)	23 (55)	49 (46)	58 (54)	0.9809
Uncertainty about the pharmacometrics of the antifungal agent in the newborn is great	22 (52)	20 (48)	63 (59)	44 (41)	0.5172
Uncertainty about the safety of the antifungal agents in the newborn is great	27 (64)	15 (36)	72 (67)	35 (33)	0.7704
Additional studies of the efficacy of the antifungal agents in the perinatal population are needed	9 (21)	33 (79)	46 (43)	61 (57)	0.0204

## Discussion

Prophylactic antifungals have been used in the NICU in preterms and high-risk newborns to reduce invasive fungal infections. The Infectious Disease Society of America (IDSA) and the European Society for Clinical Microbiology and Infectious Diseases (ESCMID) have proposed recommendations regarding this practice. The IDSA recommends the use of prophylactic antifungals in ELBW babies in NICUs with high rates of invasive candidiasis (invasive candidiasis in more than 10% of ELBW babies) [8]. The ESCMID in 2012 recommended prophylaxis in NICUs with high rates of invasive candidiasis (more than 12 % in ELBW babies). Additionally, they also proposed a risk-factor-based use of prophylaxis in ICUs with a low incidence of candidiasis (less than 2% in ELBW) [9].

According to our study, 54% of NICUs used prophylactic antifungals in neonates. Of these, half of them used antifungal prophylaxis in all ELBW babies while the other units followed a risk-factor-based approach. This was similar to a European study where 55% of NICUs used prophylactic antifungals in ELBW babies and/or high-risk babies. Data from Japan showed that a slightly lesser number of the NICUs (43%) used antifungal prophylaxis.

Studies have shown that the benefit of prophylactic antifungals is limited to NICUs with a high incidence of IFI [10,11]. Although prophylactic antifungals can reduce IFI in NICUs with a high incidence of invasive candidiasis, there was no statistically significant reduction in mortality.

The definite cut-off that can be used to identify a NICU with a high incidence of invasive candidiasis is unclear. Furthermore, in India, there is no centralized database on the incidence of IFI in NICUs. The wide variation in practice in Indian NICUs could be due to these reasons. 

O’Grady et al reported that 41% of neonatologists in the UK and Ireland used fluconazole while 53% used oral nystatin [12]. However, in our study, we observed that the first choice of prophylactic antifungal was fluconazole in a majority of the units. This was comparable to a survey from Europe including data from 28 countries where fluconazole was used in 99 of 107 units and oral nystatin and miconazole in only 3 of 107 units [4]. Very few studies on oral nystatin and miconazole exist in the literature. Most of the studies are based on fluconazole use and hence it appears to be the most preferred prophylactic agent.

In this survey, the most commonly used administration interval of fluconazole was every 72 hours. A small number of units also used 48 hours and 24-hour intervals. Earlier studies used pharmacokinetics to devise an age-based administration interval. It was later found that a 72-hour interval was equally effective [13,14]. The practice of following a 72-hour interval is consistent with current evidence and guidelines.

Prophylactic antifungals have been used in the NICU for preterms and high-risk newborns to reduce invasive fungal infections. The Infectious Disease Society of America (IDSA) and the European Society for Clinical Microbiology and Infectious Diseases (ESCMID) have proposed recommendations regarding this practice. The IDSA recommends the use of prophylactic antifungals in ELBW babies in NICUs with high rates of invasive candidiasis (invasive candidiasis in more than 10% of ELBW babies) [8]. The ESCMID in 2012 recommended prophylaxis in NICUs with high rates of invasive candidiasis (more than 12% in ELBW babies). Additionally, they also proposed a risk factor-based use of prophylaxis in ICUs with a low incidence of candidiasis (less than 2% in ELBW) [9].

According to our study, 54% of NICUs used prophylactic antifungals in neonates. Of these, half of them used antifungal prophylaxis in all ELBW babies, while the other units followed a risk factor-based approach. This was similar to a European study where 55% of NICUs used prophylactic antifungals in ELBW babies and/or high-risk babies. Data from Japan showed that a slightly smaller number of NICUs (43%) used antifungal prophylaxis.

Studies have shown that the benefit of prophylactic antifungals is limited to NICUs with a high incidence of IFI [10,11]. Although prophylactic antifungals can reduce IFI in NICUs with a high incidence of invasive candidiasis, there was no statistically significant reduction in mortality.

The definite cut-off that can be used to identify a NICU with a high incidence of invasive candidiasis is unclear. Furthermore, in India, there is no centralized database on the incidence of IFI in NICUs. The wide variation in practice in Indian NICUs could be due to these reasons.

O’Grady and Dempsey reported that 41% of neonatologists in the UK and Ireland used fluconazole, while 53% used oral nystatin [12]. However, in our study, we observed that the first choice of prophylactic antifungal was fluconazole in a majority of the units. This was comparable to a survey from Europe, which included data from 28 countries, where fluconazole was used in 99 of 107 units and oral nystatin and miconazole in only 3 of 107 units [4]. Very few studies on oral nystatin and miconazole exist in the literature. Most of the studies are based on fluconazole use, and hence it appears to be the most preferred prophylactic agent.

In this survey, the most commonly used administration interval for fluconazole was every 72 hours. A small number of units also used 48- and 24-hour intervals. Earlier studies used pharmacokinetics to devise an age-based administration interval. It was later found that a 72-hour interval was equally effective [13,14]. The practice of following a 72-hour interval is consistent with current evidence and guidelines.

Nearly half of the units used fluconazole at a dose of 3 mg/kg and others at 6 mg/kg. Previous studies showed no statistically significant difference in the reduction of IFI with either of the dosage regimens [15]. Both the IDSA and the ESCMID recommend a dose of 3-6 mg/kg. The factors that influenced the unit’s decision regarding fluconazole dose (3 or 6 mg/kg) were not studied in this survey as they do not appear clinically significant.

IDSA recommends prophylactic fluconazole in ELBW babies in intensive care units with a high rate of invasive candidiasis for six weeks. It may be given orally or intravenously. In our study, the most commonly used route was intravenous. The endpoints used to discontinue fluconazole prophylaxis were, a predetermined number of days, availability of intravenous access, and target gestational age or weight.

Based on the survey, 44% of units preferred liposomal amphotericin B, while an equal number of units preferred to use the conventional form as the therapeutic choice in IFI. Both preparations are shown to be effective [16]. Jeon et al. compared liposomal amphotericin B in 26 preterms versus conventional amphotericin B in a historical control of 20 preterms. They showed less renal toxicity but increased mortality in the liposomal arm [17]. Data from well-designed randomized controlled trials are lacking, to confirm the superiority of one over the other.

The factors that strongly influenced the non-users were the low incidence of IFI in the NICU. They were also concerned about the development of resistance. As compared to the non-users, the users felt that consensus statements from pediatric societies were needed. They also felt the need for more studies on the efficacy of antifungal agents. It may be assumed that the users felt that the development of new guidelines and new studies would rationalize and justify their practice.

One of the limitations is that we did not standardize the questionnaire used for the study. We did not adopt inclusive or random sampling while choosing the states of India that participated in the study. It was necessary to do so, as some of the smaller states have much fewer NICUs than others. However, we included two states from each of the six geographic zones of India. In our study, only one consultant from each NICU was contacted. In NICUs with no uniform protocol, the consultant’s opinion may not be entirely reflective of the unit’s practice.

## Conclusions

This study highlights variations in the practice of using prophylactic antifungals in extremely low birth weight babies and high-risk newborns in the NICU. It was observed that few NICUs use antifungals prophylactically in all babies less than 1 kg at birth, while others did not use or adopted a risk-based approach.

The choice of antifungal, dosage, and frequency of administration among the users were comparable across NICUs. The most common agent was fluconazole, administered intravenously at a dose of 3-6 mg/kg. The variations were noted in the indication and the duration of use. Evidence-based recommendations with definite criteria to define high-risk newborns will ensure uniformity in practice and standardize care.

## Appendices

Questionnaire

1. Name of Institution

2. Designation of the informant

     a. Senior consultant   b. Junior consultant   c. Senior Resident   d. Junior Resident

3. Do you use antifungal prophylaxis in all extreme preterms (<28 weeks GA or less than 1 kg birth weight)?

     a. Yes   b.   No   c. Case-based

4.  First choice of prophylactic antifungal agent in your unit is

     a. Fluconazole     b.  Amphotericin B    c.  Oral Nystatin    d. Miconazole

     e. Caspofungin    f. Combination of antifungals    g.  Not applicable

5. Most preferred dose of fluconazole (prophylaxis) in your unit is

     a. 3 mg/kg twice a week   b. 6 mg/kg twice a week   c. Others

     d. Not applicable

6. Most preferred dosing interval of fluconazole prophylaxis is

     a. 24 hours    b. 48 hours   c. 72 hours or twice weekly   d. others   e. Not applicable

7. Preferred route of administering fluconazole prophylaxis

     a. Intravenous   b. Oral   c. Based on IV access availability   d. Not applicable

8. Most preferred formulation of amphotericin B in your unit

     a. Conventional   b. Liposomal   c. Both   d. Not applicable

9. Do you use antifungal prophylaxis in any/all of the following --- tick multiple boxes if required

     a. Very low birth weight babies   b. Postnatal corticosteroids for BPD

     c. Prolonged antibiotics > 5 days   d. central venous catheter in-situ

     e. Prolonged TPN > 5 days f. Abdominal surgery   g. Not applicable

10. Discontinue prophylactic antifungals based on

     a. Pre-determined number of days   b. Availability of intravenous access   c. Till a target weight is

     attained   d. Till a target gestational age   e. Others   f. Not applicable

Grade these FACTORS based on their importance in INFLUENCING YOUR DECISION to prescribe or not to prescribe

(1-least, 5-most important)

1.    Incidence of candidiasis in your NICU is/is not high enough to justify prophylaxis

1 2 3 4 5

2.    Widespread antifungal use could lead to increased antifungal resistance

1 2 3 4 5

3.    Statement by pediatric societies in support of routine use in a subset of newborns is needed

1 2 3 4 5

4.    The criteria of high-risk patients in whom prophylaxis should be attempted need clarification

1 2 3 4 5

5.     The agent is too costly

1 2 3 4 5

 6.    The role of surveillance culture in identifying high-risk neonates needs clarification

1 2 3 4 5

7.     Uncertainty about the pharmacometrics of the antifungal agent in the newborn is great

1 2 3 4 5

8.     Uncertainty about the safety of the antifungal agents in the newborn is great

1 2 3 4 5

9.    Additional studies of the efficacy of the antifungal agents in the perinatal population are needed

1 2 3 4 5
